# Empagliflozin combined with bortezomib in the treatment of heavy and light chain amyloidosis with secondary diabetes: A case report

**DOI:** 10.1097/MD.0000000000043859

**Published:** 2025-08-15

**Authors:** Naidan Zhang, Na Zhang, Le Luo, Yiman Li, Caixia Ji, Chengliang Yuan

**Affiliations:** a Department of Clinical Laboratory, Deyang People’s Hospital, Deyang, Sichuan Province, China; b Department of Hematology, Deyang People’s Hospital, Deyang, Sichuan Province, China; c Department of Medical Records, Deyang People’s Hospital, Deyang, Sichuan Province, China.

**Keywords:** autologous stem cell transplantation, daratumumab, diabetes, empagliflozin, heavy and light chain amyloidosis

## Abstract

**Rationale:**

Primary heavy and light chain amyloidosis (AHL) is extremely rare globally. This study aimed to report the first clinical treatment of AHL secondary diabetes. We also analyzed fasting blood glucose levels across various treatments. Finally, a reference was provided for treatment and laboratory indicators in AHL with secondary diabetes.

**Patient concerns:**

The patient was treated in Department of Hematology in Deyang People’s Hospital. He underwent treatment with daratumumab (DARA) + cyclophosphamide, bortezomib, and dexamethasone (CyBorD), autologous stem cell transplantation and bortezomib for AHL, alongside empagliflozin for blood glucose management. Clinical data and laboratory results were collected during phases of treatment and follow-up.

**Diagnoses:**

This patient was a middle-aged man with 2 months of swelling in both lower limbs. Tests, including serum and urine analyses and a kidney biopsy, diagnosed him with AHL (IgA-λ).

**Interventions:**

He was diagnosed with diabetes after 4 courses of DARA + CyBorD. Then DARA was discontinued. CyBorD, autologous stem cell transplantation, and bortezomib were continued for AHL, alongside empagliflozin for blood glucose management.

**Outcomes:**

Finally, very good partial remission and effective control of blood sugar were obtained.

**Lessons:**

Current study presented a case of AHL with secondary diabetes following treatment with DARA + CyBorD. For patients with high fasting blood glucose undergoing targeted therapy or chemotherapy, oral glucose tolerance test and glycosylated hemoglobin tests were necessary for the overall health. The difference between free light chain trend changed earlier than 24-hour urine protein, offering guidance for early AHL treatment and enhancing patient quality of life.

## 1. Introduction

Heavy and light chain amyloidosis (AHL) is rare, accounting for about 4.2% of renal amyloidosis cases.^[1]^ Treatment mirrors that of light chain amyloidosis (AL), with autologous stem cell transplantation (ASCT) being the preferred method due to its effectiveness in achieving hematological remission and extending survival. Daratumumab (DARA) is a humanized IgG1-κ antibody targeting the CD38 antigen.^[2]^ Phase III clinical trials have shown that DARA, in combination with cyclophosphamide, bortezomib and dexamethasone (CyBorD), enhances the overall response rate, organ response rate and progression-free survival.^[3]^ However, increased use of targeted therapies can lead to immune-related adverse events, such as diabetic ketosis and diabetic ketoacidosis.^[4]^ It’s unclear if DARA causes diabetes in AHL patients. Current study report the diagnosis and treatment of an AHL patient with diabetes. The patient underwent treatment with DARA+CyBorD, ASCT and bortezomib for AHL, alongside empagliflozin for blood glucose management, ultimately achieving very good partial response (VGPR) and effective glycemic control, as detailed below.

## 2. Case report

In March 2022, a middle aged man visited the Nephrology Department with 2 months of swelling in both lower limbs. Tests, including serum and urine immunofixation electrophoresis and a kidney biopsy, diagnosed him with AHL (IgA-λ; Supplemental Files 1–3, Supplemental Digital Content, https://links.lww.com/MD/P686). Initial lab results showed fasting blood glucose (FBG) of 6.54 mmol/L, no urine glucose, urine protein 4+, and 24-hour urine protein (24hUP) at 7.73 g/L. The serum free light chain difference was 4.07 mg/L. After 4 DARA+CyBorD treatment cycles, his FBG rose to 8.91 mmol/L, with postprandial levels of 10.90 mmol/L and 12.42 mmol/L. Diabetes-related antibody tests were negative. Urine glucose stayed negative, urine protein dropped to 2+, and 24hUP was 6.29 g/L. He was diagnosed with a specific diabetes type, leading to stopping DARA. After 4 more CyBorD courses, the patient’s FBG was 6.29 mmol/L, urine glucose was negative, difference between free light chain (dFLC) was 1.13 mg/L, and 24hUP was 3.49 g/L, indicating a partial response (PR). He was monitored every 3 months.

In March 2023, he was hospitalized for weakness, with indicators showing disease progression: 24hUP at 5.71 g/L and positive IgA and λ chains. He was admitted for ASCT, received melphalan pretreatment, and a total of 297 mL CD34+stem cells (153.82 × 10^9^/L) were transfused. One month after the ASCT, FBG was 6.09 mmol/L and 24hUP was 4.84 g/L. CyBorD continued for 3 months, again showing PR. His FBG ranged from 5.16 to 7.52 mmol/L without hypoglycemic medication.

**Figure 1.**
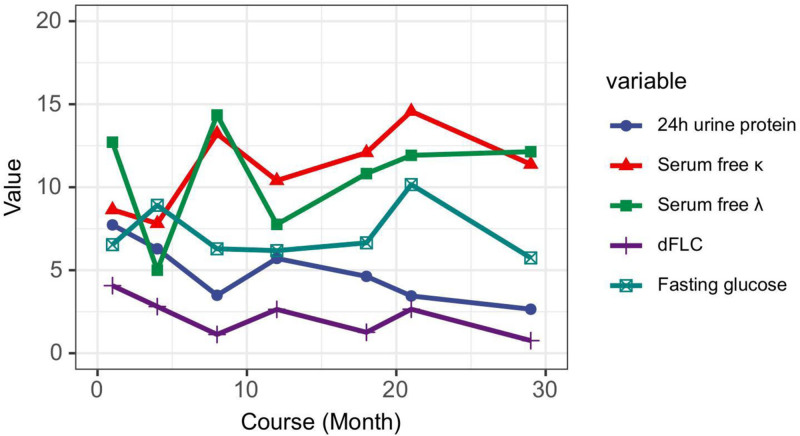
The trend of laboratory indicators in the course of AHL. The *x*-axis shows the disease duration in months, while the *y*-axis displays the lab index value. The figure indicates that dFLC closely aligns with 24-h urinary protein levels. Fasting blood glucose first rose after 4 DARA+CyBorD courses and again 9 mo post-ASCT transplantation. AHL = heavy and light chain amyloidosis, dFLC = difference between free light chain, DARA = daratumumab, CyBorD = cyclophosphamide, bortezomib and dexamethasone, ASCT = autologous stem cell transplantation.

In December 2023, the patient had an FBG of 10.16 mmol/L and glycosylated hemoglobin (HbA1c) of 7.7%, with elevated serum free κ and λ chains. The dFLC was 2.65 mg/L. Empagliflozin (10 mg/q d) was prescribed to lower FBG, and bortezomib was used for posttransplant maintenance. After 9 months, FBG dropped to 5.74 mmol/L and HbA1c to 6.5%, showing effective glycemic control. For AHL, dFLC was 0.76 mg/L, 24hUP was 2.65 g/L, serum IgA chain was weakly positive, and the λ chain was negative. After nearly 30 months, AHL was assessed as VGPR. Clinical data were in Table 1 and Figure 1.

**Table 1:** Changes of laboratory indexes with clinical treatments.

Treatment (time)	First visit (March 2022)	After DARA+CyBorD (June 2022)	After CyBorD (November 2022)	Pre ASCT (March 2023)	After ASCT (September 2023)	Before empagliflozin+bortezomib (December 2023)	After empagliflozin+bortezomib (September 2024)
24hUP (g/L)	7.73	6.29	3.49	5.71	4.63	3.45	2.65
Creatinine (µmol/L)	69.8	66.0	62.1	76.2	62.8	64.6	55.9
IFE-IgA	+	−	−	+	+	Weak +	Weak +
IFE-λ	+	−	−	Weak +	Weak +	−	−
Serum free κ (mg/L)	8.64	7.81	13.22	10.40	12.08	14.57	11.38
Serum free λ (mg/L)	12.71	5.00	14.35	7.76	10.82	11.92	12.14
dFLC (mg/L)	4.07	2.81	1.13	2.64	1.26	2.65	0.76
κ/λ	0.68	1.56	0.92	1.34	1.12	1.22	0.93
FBG (mmol/L)	6.54	8.91	6.29	6.18	6.65	10.16	5.74
Urine glucose	−	−	−	−	1+	2+	3+
Urine protein	4+	2+	3+	3+	3+	1+	2+
Total protein (g/L)	49.7	42.9	45.3	53.7	57.7	57.9	61.0
Albumin (g/L)	25.4	28.4	31.5	38.5	40.7	39.9	41.2
A/G	1.05	1.96	2.28	2.53	2.39	2.22	2.1
Serum albumin (%)	51	67.2	63.1	68.3	67.9	66.2	63.9
α_1_ (%)	4.7	4.2	4.2	4.0	3.8	4.3	4
α_2_ (%)	15.4	15.4	14.6	11.4	9.0	10.1	9.3
β_1_ (%)	6.7	5.5	5.5	5.6	5.8	6.7	6.4
β_2_ (%)	15.4	4.4	5.3	4.9	4.7	4.1	5.6
γ (%)	6.8	3.3	7.3	5.8	8.8	8.6	10.8
M protein (%)	14.7	0.0	0.00	0.00	0.00	0.00	0.00

24hUP = 24-hour urine protein, ASCT = autologous stem cell transplantation, CyBorD = cyclophosphamide, bortezomib and dexamethasone, DARA = daratumumab, dFLC = difference between free light chain, FBG = fasting blood glucose, IFE = immunofixation electrophoresis.

**Figure 2.**
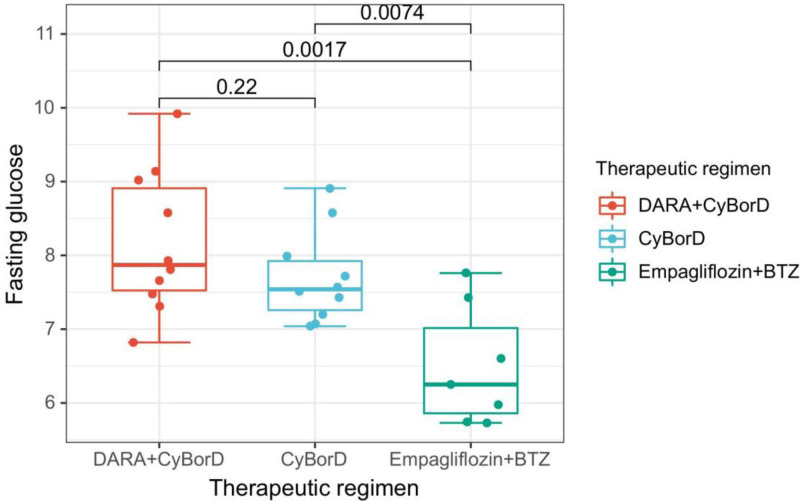
Fasting blood glucose in different treatment groups. No significant difference in fasting blood glucose between DARA+CyBorD and CyBorD (*P* = .22). However, levels of fasting blood glucose differed between DARA+CyBorD and empagliflozin+bortezomib (*P* = .0017), and between CyBorD and empagliflozin+bortezomib (*P* = .0074). DARA = daratumumab, CyBorD = cyclophosphamide, bortezomib and dexamethasone, BTZ = bortezomib.

To investigate the link between targeted/chemotherapy treatments and FBG levels, we divided results into 3 groups: DARA+CyBorD, CyBorD, and empagliflozin+bortezomib. FBG was measured from the second day to the last day of treatment. Inclusion criteria were: from the day after treatment started to its end; venous blood samples collected without anticoagulants; consistent use of the same instruments and reagents. Exclusion criteria were: FBG readings from non-targeted treatments or the first day were excluded; FBG measurements on days involving glucose-affecting drugs or insulin were excluded; fingertip blood glucose readings were excluded. A *T*-test was used for statistical analysis. The study found no significant difference in FBG between DARA+CyBorD and CyBorD (*P* = .22). However, FBG differed significantly between DARA+CyBorD and empagliflozin+bortezomib (*P* = .0017), and between CyBorD and empagliflozin+bortezomib (*P* = .0074). These results implied that DARA+CyBorD did not significantly elevate FBG in AHL patients compared to CyBorD alone (Supplemental File 4, Supplemental Digital Content, https://links.lww.com/MD/P686). Empagliflozin effectively reduced hyperglycemia in patients with elevated FBG undergoing targeted and chemotherapeutic treatments, with data shown in Figure 2. The patient’s blood lipid levels in the entire treatment had no significant changes (Supplemental File 5, Supplemental Digital Content, https://links.lww.com/MD/P686).

## 3. Discussion

There is limited research on AHL, mainly addressing clinical variations, prognosis, and pathology. Documentation on diagnosing, treating, and monitoring AHL with diabetes is scarce. In this study, we presented the first clinical treatment case of AHL (IgA-λ) with diabetes, where the patient achieved VGPR and controlled FBG after 30 months. We identified 2 key points for clinicians from the treatment review.

For patients with abnormal FBG levels, comprehensive evaluations like the oral glucose tolerance test (OGTT) were recommended before starting targeted therapy or chemotherapy. In this case, the AHL diagnosis was clear, with only mild kidney damage.^[5]^ This AHL classification indicated a clear diagnosis and favorable prognosis. However, the slightly elevated FBG was overlooked before DARA+CyBorD treatment. Since the patient had no diabetes history and the FBG level wasn’t diagnostic, it was managed with lifestyle and dietary changes. It was unclear if DARA caused diabetes, as our literature review found no evidence of DARA inducing diabetes. FBG levels were analyzed during treatment and no significant differences were found between DARA+CyBorD and CyBorD groups. Research indicated that dexamethasone temporarily raised FBG levels, which normalized after stopping the medication.^[6,7]^ In this study, the patient receiving DARA+CyBorD experienced elevated FBG, which returned to pretreatment after discontinuing DARA. The patient did not show severe diabetes-like symptoms, suggesting DARA may raise FBG but wasn’t a direct cause of diabetes. Monitoring FBG in patients before targeted therapies is important, and OGTT can provide a comprehensive health assessment.

To provide effective treatment for AHL with diabetes, we suggested assessing AHL and diabetes separately. AHL treatment should follow established guidelines, while diabetes management should focus on better glycemic control and quality of life. In this study, DARA, CyBorD, ASCT and bortezomib were used for AHL treatment. Twenty four hUP, serum immunofixation electrophoresis, and dFLC levels were monitored treatment progress. The dFLC trend generally matched the 24hUP. Notably, after ASCT, dFLC rose post-withdrawal while urine protein declined. Bortezomib administration led to a consistent decrease in both dFLC and urine protein, with dFLC changes appearing earlier. Previous research showed that a decrease in dFLC in AL patients reliably predicted complete hematologic response, with 87.5% sensitivity and 81.3% specificity.^[8]^ Another study found that a dFLC below 10 mg/L was linked to longer overall survival and extended time to next treatment in AL.^[9]^ We proposed that tracking dFLC trends during the PR phase provided valuable insights for early intervention in AHL, potentially reducing organ involvement.

After ensuring the treatment of AHL in this patient, it was crucial to select an appropriate antidiabetic drug. Empagliflozin and dapagliflozin, 2 oral hypoglycemic agents with distinct mechanisms, have been instrumental in the personalized management of type 2 diabetes mellitus. Recent research indicates that either empagliflozin or dapagliflozin have comparable long-term kidney outcomes.^[10]^ The guidelines of the International Society of Amyloidosis indicated that for amyloidosis patients with proteinuria, the addition of sodium-glucose cotransporter 2 inhibitors could be considered.^[11]^ In this case, empagliflozin was the preferred agent. It was based on several factors. First, the patient was middle aged, with long-term impaired FBG levels as the main character. Second, the patient exhibited proteinuria, with an estimated glomerular filtration rate ≥20 mL/min/1.73 m². Most importantly, the patient had a background of AHL, which was prone to cause cardiovascular damage. This was the most significant complication affecting the prognosis of patients. Empagliflozin offered long-term benefits, including enhanced myocardial energy metabolism and anti-inflammatory effects.

Following communication with the patient regarding the potential risk of urinary infection, the patient commenced treatment with empagliflozin. Initial laboratory results indicated a consistent strong positive result of urine glucose. After a 9-month treatment, FBG returned to normal, and HbA1c decreased by 1.2%. Diabetes-related symptoms have been effectively managed. What’s more, this patient did not present with atherosclerotic cardiovascular disease. His liver and kidney functions, as well as the body mass index, were generally normal.

Empagliflozin was administered for glycemic control. Empagliflozin, a sodium-glucose cotransporter 2 inhibitor, was used for glycemic control, promoting glucose excretion through urine by reducing kidney reabsorption. It effectively lowered HbA1c and FBG levels and reduced the risk of kidney disease, mainly treating type 2 diabetes in adults.^[12–14]^ While it helped manage FBG levels during treatments, it could increase the risk of urinary tract infections.^[15]^ Although the patient showed no symptoms, potential risks should be monitored with the continuation of treatment.

This study also has limitations. Before undergoing DARA treatment, the patient was not tested for items including diabetes autoantibodies. This led to our inability to clearly identify the cause of the patient’s elevated FBG before treatment. As AHL is a rare disease, more data are needed to support the long-term effects of immune-targeted therapy on blood glucose in AHL.

## 4. Conclusion

Current study presented a case of AHL with high FBG following treatment with DARA + CyBorD. For patients with high FBG undergoing targeted therapy or chemotherapy, OGTT and HBA1c tests were necessary for the overall health. The dFLC trend changed earlier than 24hUP, offering guidance for early AHL treatment and enhancing patient quality of life.

## Acknowledgments

We appreciated the patient’s persistence in follow-up. And we wish him a good condition to face life.

## Author contributions

**Data curation:** Naidan Zhang, Na Zhang, Le Luo.

**Conceptualization:** Chengliang Yuan.

**Formal analysis:** Naidan Zhang.

**Funding acquisition:** Naidan Zhang.

**Investigation:** Naidan Zhang, Na Zhang.

**Methodology:** Yiman Li, Caixia Ji, Chengliang Yuan.

**Project administration:** Naidan Zhang, Chengliang Yuan.

**Resources:** Na Zhang.

**Supervision:** Le Luo.

**Software:** Naidan Zhang.

**Writing – original draft:** Naidan Zhang.

**Writing – review & editing:** Naidan Zhang, Chengliang Yuan.

## Supplementary Material


