# Supplementary material for: Empagliflozin combined with bortezomib in the treatment of heavy and light chain amyloidosis with secondary diabetes: A case report
Source: Medicine (Baltimore). 2025 Aug 15;104(33):e43859. doi: 10.1097/MD.0000000000043859 (PMC12367034; doi:10.1097/MD.0000000000043859)
Supplement: Supplementary file 1 [file medi-104-e43859-s001.docx]

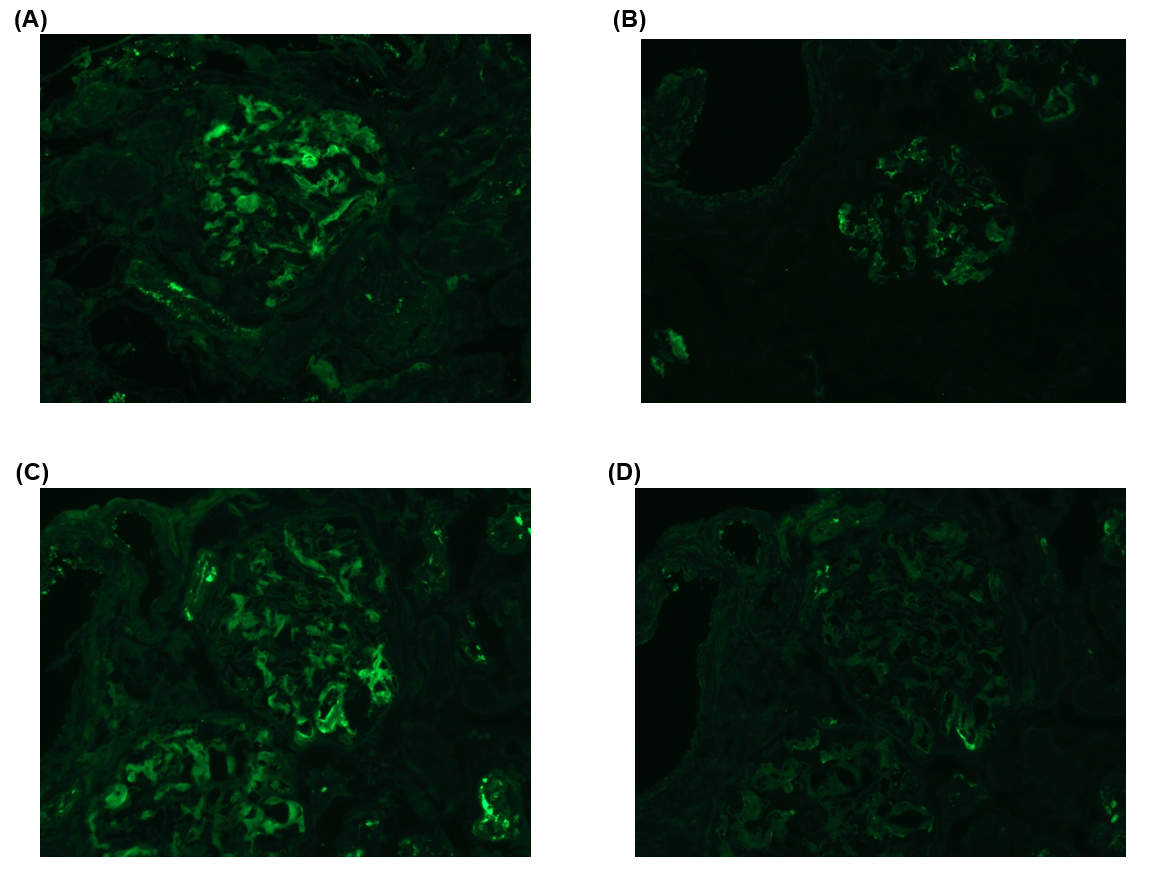


**Supplemental file 1** Illustrates immunofluorescence staining of kidney frozen sections. Sixteen markers were assessed, including IgG, IgA, IgM, C3, C1q, Fib, ALB, κ, λ, IgG1, IgG2, IgG3, IgG4, PLA2R, THSD7A, and AA. Results showed IgA (2+), IgM (+), and λ (+). IgA deposits were observed along the glomerular capillary loops, mesangial area, and renal interstitium, with amyloid κ-negative and λ-positive findings. Panels depict: (**A**) IgA, (**B**) IgM, (**C**) λ, and (**D**) κ.

**Abbreviation:** Ig, immunoglobulin; Fib: fibrinogen; ALB, albumin; PLA2R, phospholipase A2 receptor; THSD7A, thrombospondin type I domain containing protein 7A, AA: serum amyloid A.


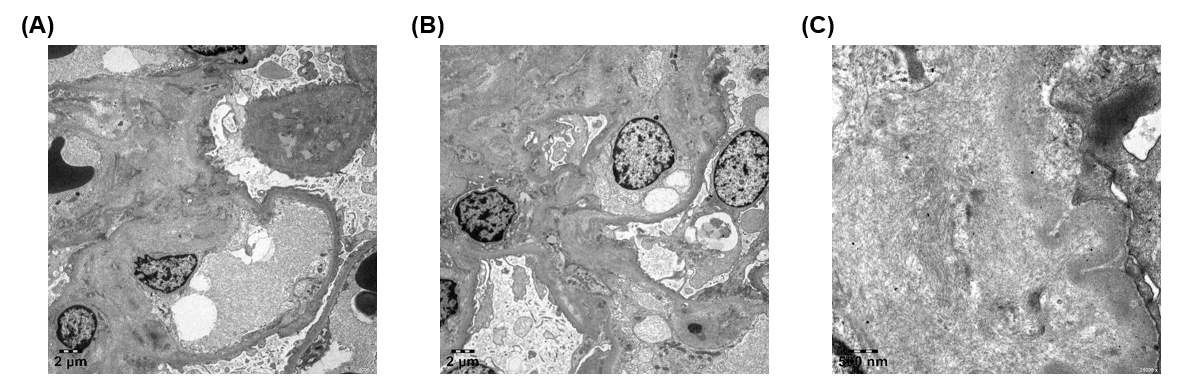


**Supplemental file 2** Ultraelectron microscope image of renal tissue showed significant changes. The glomerulus exhibited vacuolar degeneration in capillary endothelial cells, segmental thickening of the basement membrane, and vacuolar degeneration with diffuse foot process fusion in visceral epithelial cells. There was extensive deposition of fibrous material in the renal interstitium, arterioles, mesangial region, and basement membrane, appearing rigid, unbranched, and disordered.


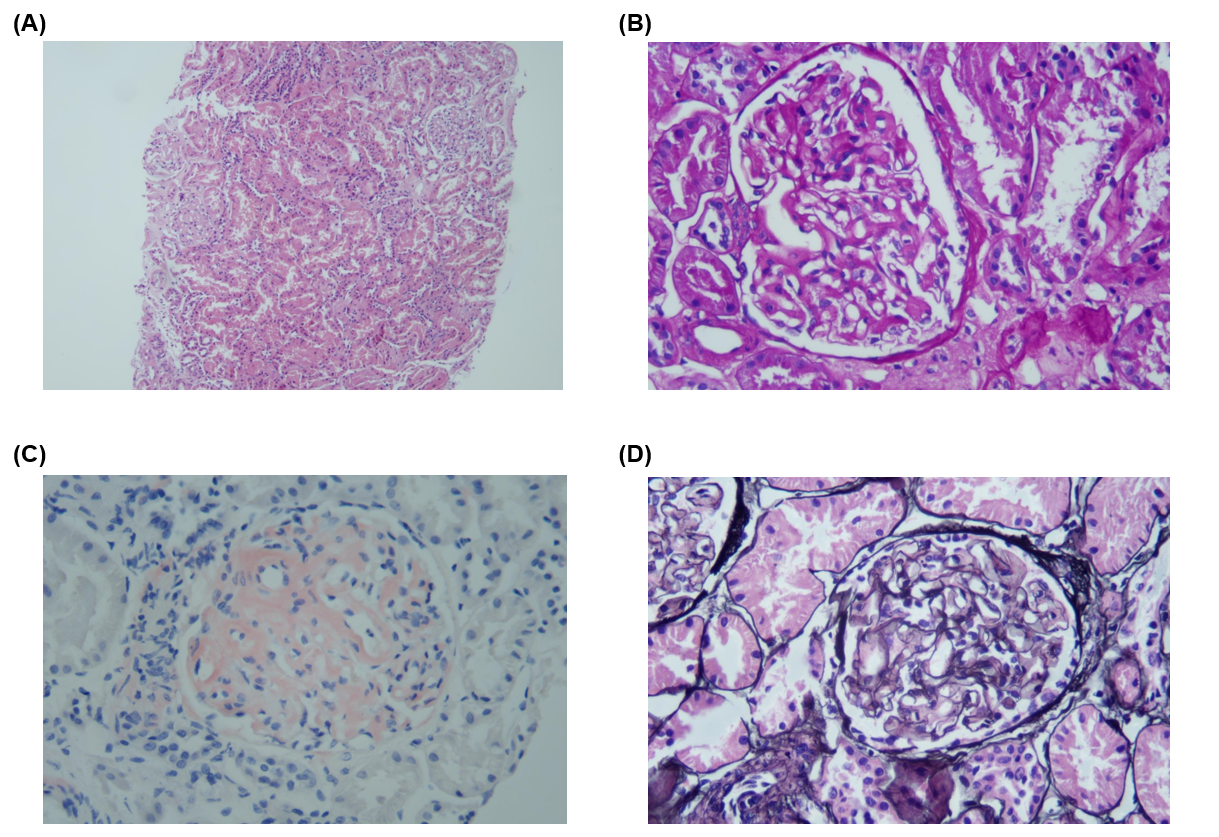


**Supplemental file 3** Renal tissue microscopy with HE, PAS, PASM, and Masson. The results showed powdery, homogeneous material in the mesangial area, compressed capillary loops, and similar deposits in the renal interstitial and arteriole walls. Congo red staining was positive. (**A**) HE; (**B**) PAS; (**C**) Congo red; (**D**) PASM+Masson.

**Abbreviation:** HE, hematoxylin-eosin staining; PAS, periodic acid-Schiff staining; PASM, periodic-acid silver methenamine.

**Supplemental file 4** Fasting blood glucose in different treatment groups

| **Therapeutic regimen** | **Fasting glucose (mmol/L)** |
| --- | --- |
| DARA+CyBorD | 9.14 |
| DARA+CyBorD | 7.66 |
| DARA+CyBorD | 9.92 |
| DARA+CyBorD | 9.02 |
| DARA+CyBorD | 7.81 |
| DARA+CyBorD | 7.31 |
| DARA+CyBorD | 7.48 |
| DARA+CyBorD | 8.58 |
| DARA+CyBorD | 7.93 |
| DARA+CyBorD | 6.82 |
| CyBorD | 8.58 |
| CyBorD | 7.99 |
| CyBorD | 7.57 |
| CyBorD | 7.20 |
| CyBorD | 7.72 |
| CyBorD | 7.51 |
| CyBorD | 7.43 |
| CyBorD | 7.07 |
| CyBorD | 7.04 |
| CyBorD | 8.91 |
| Empagliflozin+BTZ | 7.43 |
| Empagliflozin+BTZ | 5.73 |
| Empagliflozin+BTZ | 6.25 |
| Empagliflozin+BTZ | 6.60 |
| Empagliflozin+BTZ | 7.76 |
| Empagliflozin+BTZ | 5.98 |
| Empagliflozin+BTZ | 5.74 |

**Abbreviation**: DARA, daratumumab; CyBorD, cyclophosphamide, bortezomib and dexamethasone; BTZ, bortezomib.

**Supplemental file 5** The patient's renal function and blood lipid levels in the entire treatment

| **Treatment (Time)** | **First visit (2022.3)** | **After DARA+CyBorD (2022.6)** | **After CyBorD (2022.11)** | **Pre ASCT (2023.3)** | **After ASCT (2023.9)** | **Before Empagliflozin + bortezomib (2023.12)** | **After Empagliflozin+ bortezomib (2024.9)** |
| --- | --- | --- | --- | --- | --- | --- | --- |
| **Renal function** |  |  |  |  |  |  |  |
| Creatinine (umol/L) | 69.8 | 66.0 | 62.1 | 76.2 | 62.8 | 64.6 | 55.9 |
| Urea (mmol/L) | 5.70 | 5.92 | - | 3.81 | 3.92 | 3.50 | - |
| Uric acid (umol/L) | 417.0 | 526.7 | - | 519.7 | 513.2 | 409.3 | - |
| **Blood lipid levels** |  |  |  |  |  |  |  |
| Total cholesterol (mmol/L) | - | 10.45 | - | 7.45 | 5.26 | 8.62 | - |
| Triglyceride (mmol/L) | - | 5.78 | - | 3.23 | 2.60 | 6.07 | - |
| HDL-C (mmol/L) | - | 0.65 | - | 1.64 | 1.32 | 1.02 | - |
| LDL-C (mmol/L) | - | 4.50 | - | 5.17 | 2.58 | 4.54 | - |
